# Biological and genetic characterization of a newly established human external auditory canal carcinoma cell line, SCEACono2

**DOI:** 10.1038/s41598-023-46926-y

**Published:** 2023-11-10

**Authors:** Noritaka Komune, Kuniaki Sato, Mayumi Ono, Akira Imaizumi, Shogo Masuda, Shinsaku Itoyama, Tomomi Manako, Ryosuke Kuga, Takahiro Hongo, Ryunosuke Kogo, Hideya Onishi, Takashi Nakagawa

**Affiliations:** 1 Department of Otorhinolaryngology, Graduate School of Medical Sciences, Kyushu University, 3-1-1 Maidashi Higashi-Ku, Fukuoka, 812-8582 Japan; 2 Department of Cancer Therapy and Research, Graduate School of Medical Sciences, Kyushu University, 3-1-1 Maidashi Higashi-Ku, Fukuoka, 812-8582 Japan

**Keywords:** Cancer genomics, Head and neck cancer, Cellular microbiology, Cell culture

## Abstract

Squamous cell carcinoma of the external auditory canal (EACSCC) is an extraordinarily rare and aggressive malignant disease. Establishment of EACSCC cell line with robust molecular characteristics is essential for the basic and translational research of EACSCC. Here, we show the newly established EACSCC cell line SCEACono2, derived from a patient with well-to-moderately differentiated EACSCC. We analyzed histologic and genetic features of SCEACono2 hiring multiple experiments, including next-generation sequencing (NGS). Immunocytochemical staining of SCEACono2 showed positivity of p53 and SCC1/2. Furthermore, SCEACono2 exhibited a unique characteristic that cytokeratin, vimentin as well as cancer stem cell markers (CD44, CD133, ALP and Oct3/4) were positive. SCEACono2 had an ability to form tumors at the temporal lesion xenograft nude mice model. NGS revealed that SCEACono2 harbored the somatic mutations of *TP53* (p.G245S) and *NOTCH1* (p.A465T). RNA-seq and downstream bioinformatics analysis revealed significant enrichment of genes involved in inflammation and cell adhesion in SCEACono2 compared to SCC-9 and HSC-4. STR profiling indicated no evidence of cross-contamination. In conclusion, SCEACono2 could serves as a promising and robust research resource of EACSCC in vitro and in vivo.

## Introduction

Squamous cell carcinoma of the external auditory canal is extraordinarily rare^1–4^. The external auditory canal is divided into bony and cartilaginous parts. Both parts are covered with cutaneous tissue, and the tympanic membrane is at the most medial part. Chronic inflammation produced especially by frequent ear-picking can be caused carcinogenesis in the skin covering the bone and cartilaginous part of the external auditory canal^5^. However, its rarity makes it difficult to set up prospective clinical studies and complicates retrospective meta-analysis, preventing the accumulation of high-quality evidence. Furthermore, the delay in establishing the cell line causes the lack of basic research evidence. So far, few reports of molecular biological studies on external auditory canal carcinoma have been reported. Almost studies have mainly used the histopathological/immunohistochemistrical method on clinical samples^6–11^.

The establishment of the stable cell line of the external auditory canal carcinoma, which can be the most crucial tool for the experiments both in vivo and in vitro, has been desired to accumulate the evidence of molecular biology study. A stable cell line of this carcinoma is mandatory to reveal the precise molecular characterization of external auditory canal squamous cell carcinoma and proceed with the molecular biological experiment both in vivo and in vitro. Now reported cell line of external auditory canal squamous cell carcinoma was established by Mario A. Hermsen’s group in Spain^12^. Until now, there has not been a report on the cell line derived from the Asian population. In Japan, ear picking, also called “mimikaki”, is a popular habit and a unique culture, which has been considered a risk factor for chronic inflammation leading to the carcinogenesis of the external auditory canal carcinoma^5^. Thus, we have tried to establish the stable cell line of the external auditory canal from Japanese people. Sekino et al.^13^ established the method to maintain the primary culture of the external auditory SCC derived from Japanese. We finally established the stable cell line of this carcinoma derived from a Japanese patient using this protocol. Furthermore, we analyze the biological and genetic characteristics of this stable cell line in this study to demonstrate the value of this cell line as a tool for molecular biology research.

## Results

### Establishment of SCEACono2 derived from a primary external auditory canal carcinoma tissue

**Figure 1:**
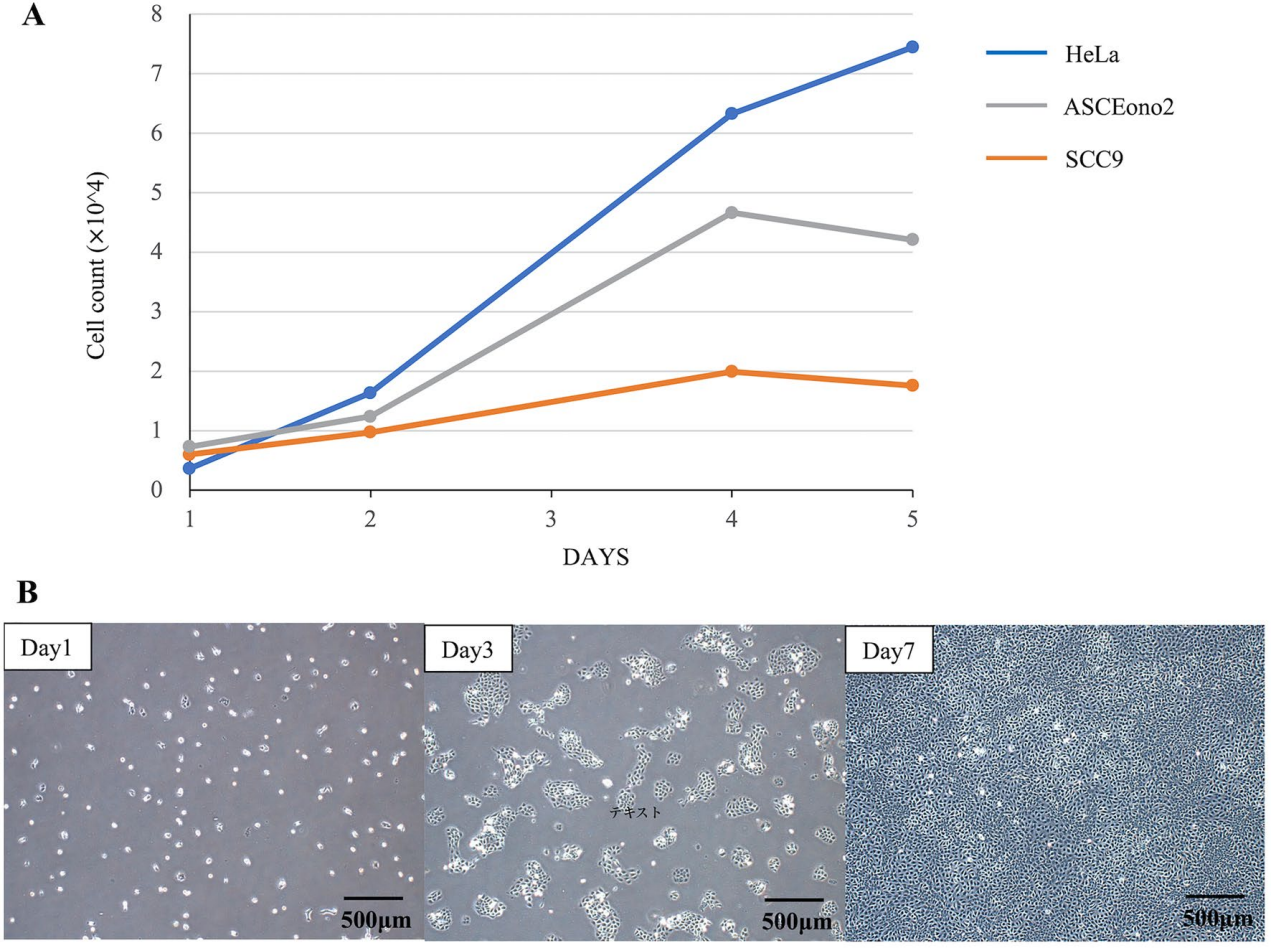
Proliferative ability of SCEACono2 cells. (**A**) Growth curve of SCEACono2 cells in 5 days. (**B**) Growth of SCEACono2 cells at 2, 4, and 5 days after seeding.

The patient was a 71-year-old woman with well-to-moderately differentiated SCC of the right external auditory canal (clinical stage: cT4N0M0). Tumor tissue was collected from surgically resected primary site. Then, the primary cells derived from the tumor tissue cultured following the previously reported method^13^. We yielded a clonally proliferating tumor colony on a feeder layer of fibroblast cells (MMC-TIG-1-20). Subsequently, the tumor colonies were picked up and seeded on culture dishes without the feeder cells. Among the picked-up colonies, we have selected the colony that survived and grew up as a monolayer on a dish, which is presumably suitable for establishment of a cell line. To date, these cells have been passaged in vitro for more than 50 passages and exhibited stable growth and thus we named this stable cell line as SCEACono2. The population doubling time of this cell line was about 24 h (Fig. 1A). SCEACono2 adhered firmly to culture flask surface, forming island-like colonies, and had epithelial-like morphology (Fig. 1B). Importantly, the established cell line did not exhibit signs of senescence, which is considered immortalized. Any signs of contamination such as fungus, bacteria or mycoplasma was not evident in the cultured SCEACono2 cells.

### SCEACono2 is positive for SCC markers and cancer stem cell markers

**Figure 2:**
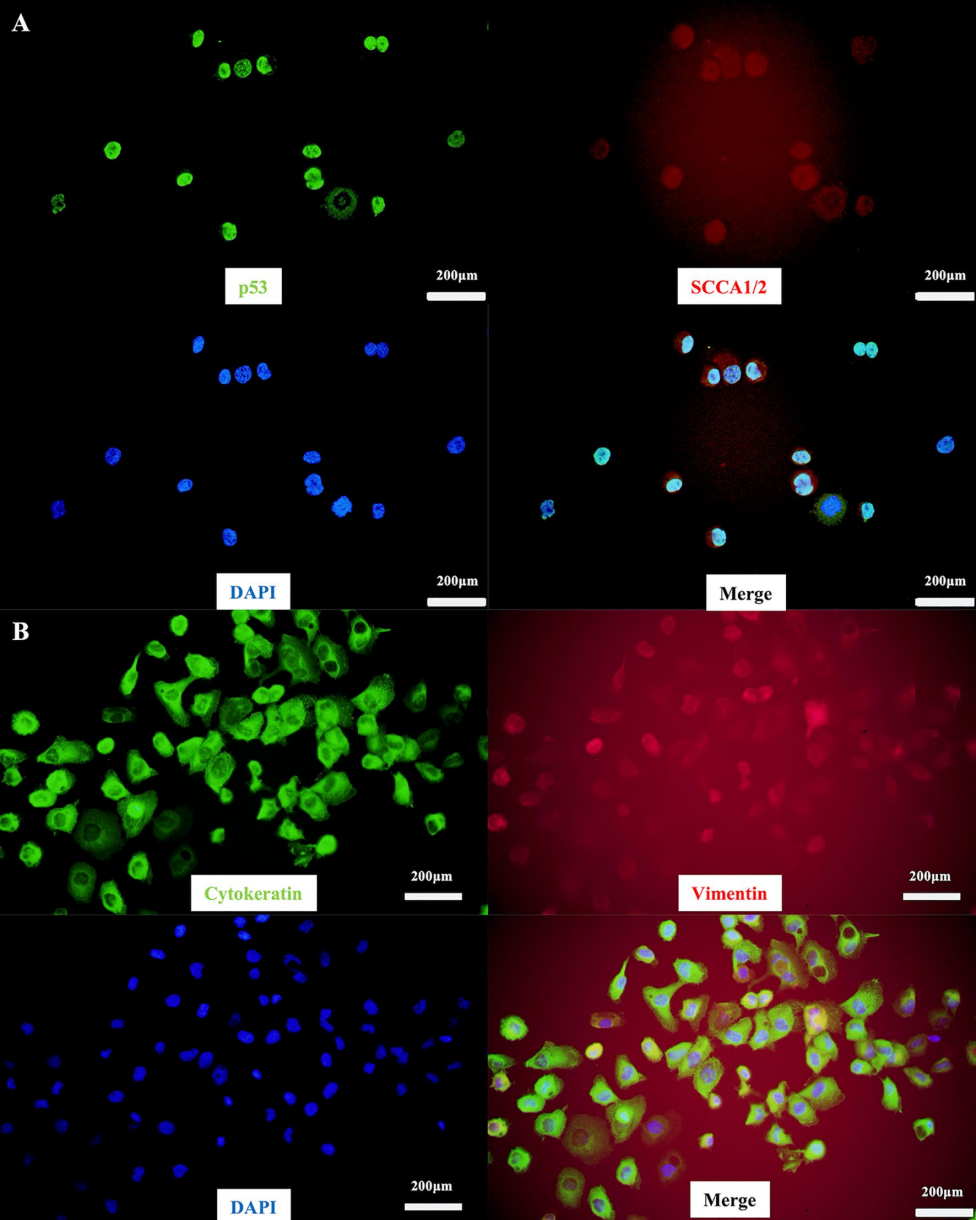
Immunostaining of SCEACono2 cell. (**A**) Fluorescence immunostaining of SCEACono2 cells with cancer-specific antibodies. The cells were double-stained with [anti-p53-Alexa 488/green] and [anti-SCCA1/2 + Alexa Fluor® 555 anti-mouse IgG/red]. The SCEACono2 cells have double positivity for p53 and SCC. (**B**) Fluorescence immunostaining of SCEACono2 cells with anti-cytokeratin-Alexa 488 (green) and anti-vimentin-PE (red). SCEACono2 cells were positive for both [anti-pan-Cytokeratin-Alexa 488/green] and [anti-Vimentin-PE/red]. Cytokeratin and vimentin are located in the cytoplasm but not in nuclei.

**Figure 3:**
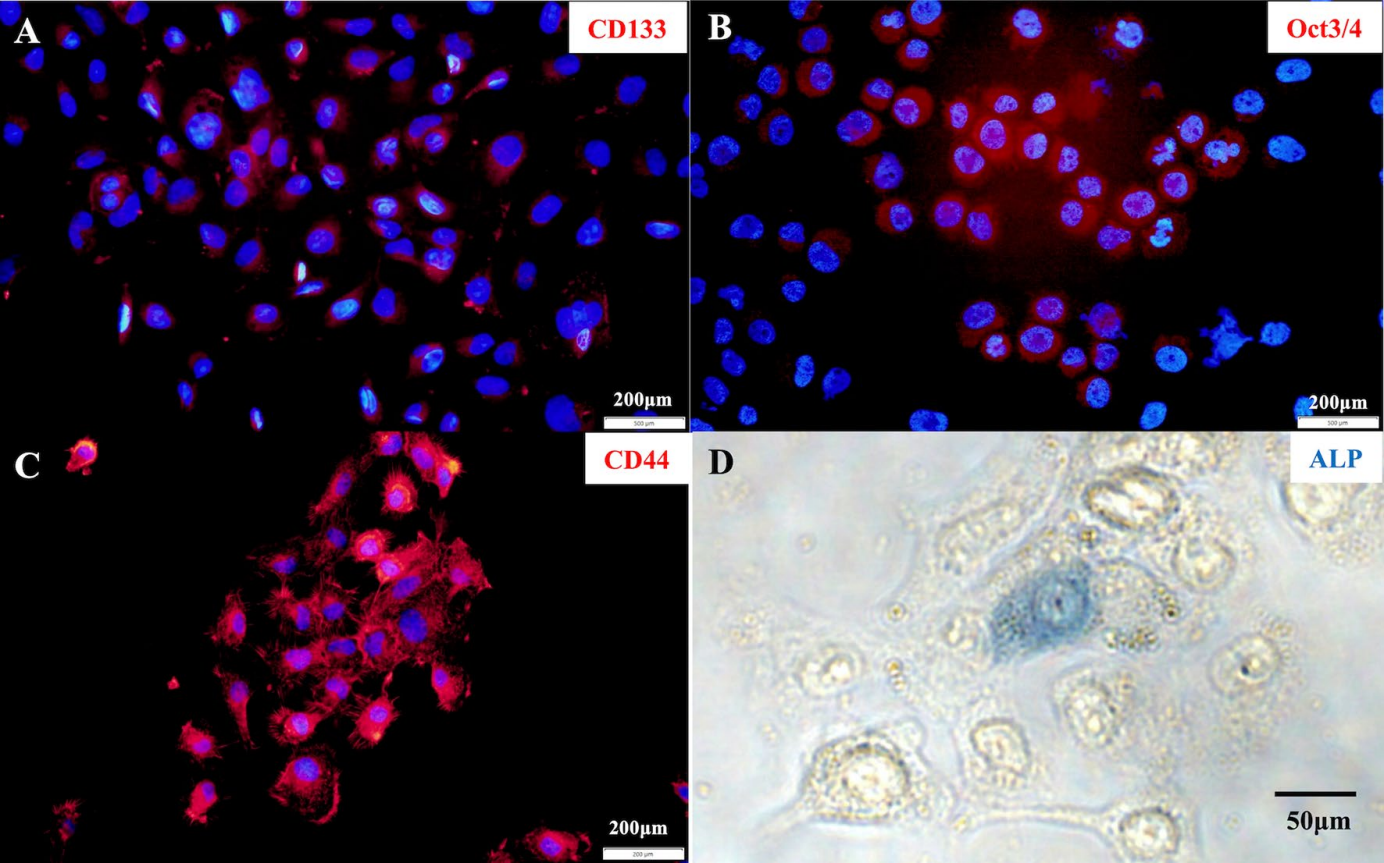
Immunofluorescence and alkaline phosphatase staining in SCEACono2 cell. (**A**–**C**) SCEACono2 cell was tested with anti-CD44-PE (**A**), anti-CD133-APC (**B**), and anti-Oct-3/4 (**C**) + Alexa Fluor® 555 anti-mouse IgG. DAPI (blue) was used to identify the location of cell nuclei for immunofluorescence staining. SCEACono2 cells have positivity of CD44, CD133, or Oct3/4. (**C**) ALP activity is partially observed in SCEACono2 cells.

We next validated the characteristics of SCEACono2 as a SCC-derived cell line. For this purpose, we performed immunocytochemical staining of SCC marker proteins. SCEACono2 exhibited positivity for p53 and SCCA1/2 (Fig. 2A). Furthermore, both cytokeratin and vimentin were positive, suggesting a hybrid state of epithelial and mesenchymal characteristics (Fig. 2B). In order to examine stemness-like features of SCEACono2 cells, the presence of cancer stem cell markers (CD44, CD133, Oct3/4, ALP) was examined^14–17^. SCEACono2 cells showed positive for all of these cancer stem cell markers (Fig. 3A–D).

### Chromosomal instability of SCEACono2 cell line

**Figure 4:**
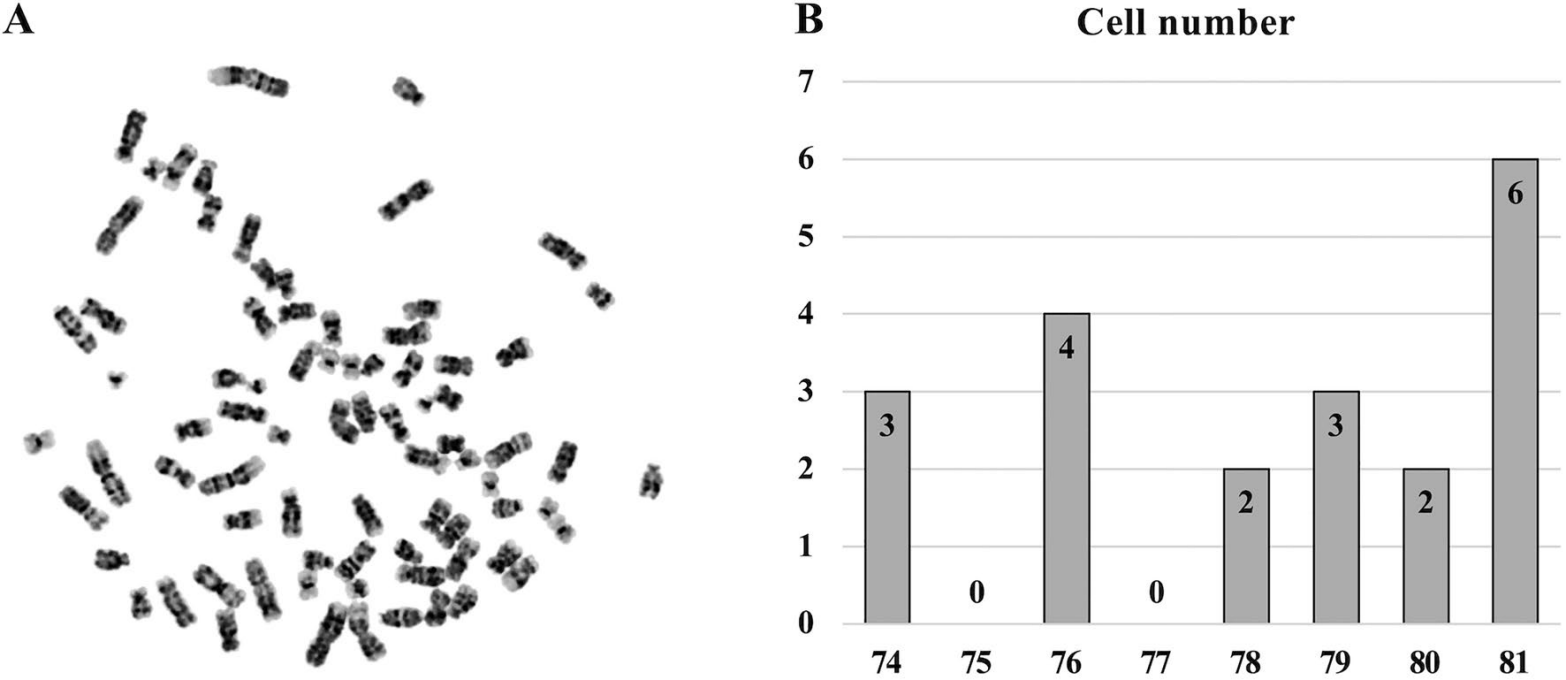
Chromosomal numbers of SCEACono2 cells. (**A**) The photograph represents Giemsa-stained metaphase chromosomes from SCEACono2 cells. (**B**) Chromosome number analysis was performed on 20 cells. The number of chromosomes varied from 74 to 81, with 81 chromosomes being the most common (6 cells).

The complicated karyotype and abnormal chromosome number of the SCEACono2 cells were revealed by chromosome analysis (Fig. 4A). Chromosome number analysis was performed on 20 cells. The number of chromosomes varied from 74 to 81, with 81 being the most common (6 cells) (Fig. 4B). Among these cells, karyotyping analysis was also performed on 8 cells that could be analyzed. Eight cells showed abnormal karyotypes, indicating chromosomal instability. We observed the five most common chromosomal aberrations in these cells as follows;

1. A chromosome with an additional fragment of unknown origin on the short arm of chromosome 9.
2. A chromosome with an additional fragment of unknown origin on the short arm of chromosome 14.
3. A chromosome with an additional fragment of unknown origin on the long arm of chromosome 18.
4. Marker chromosome 1 (mer 1).
5. Marker chromosome 3 (mer 3).

The karyograms of each cell are shown in Supplementary Fig. 1.

### The short tandem repeat (STR) profile

**Figure 5:**
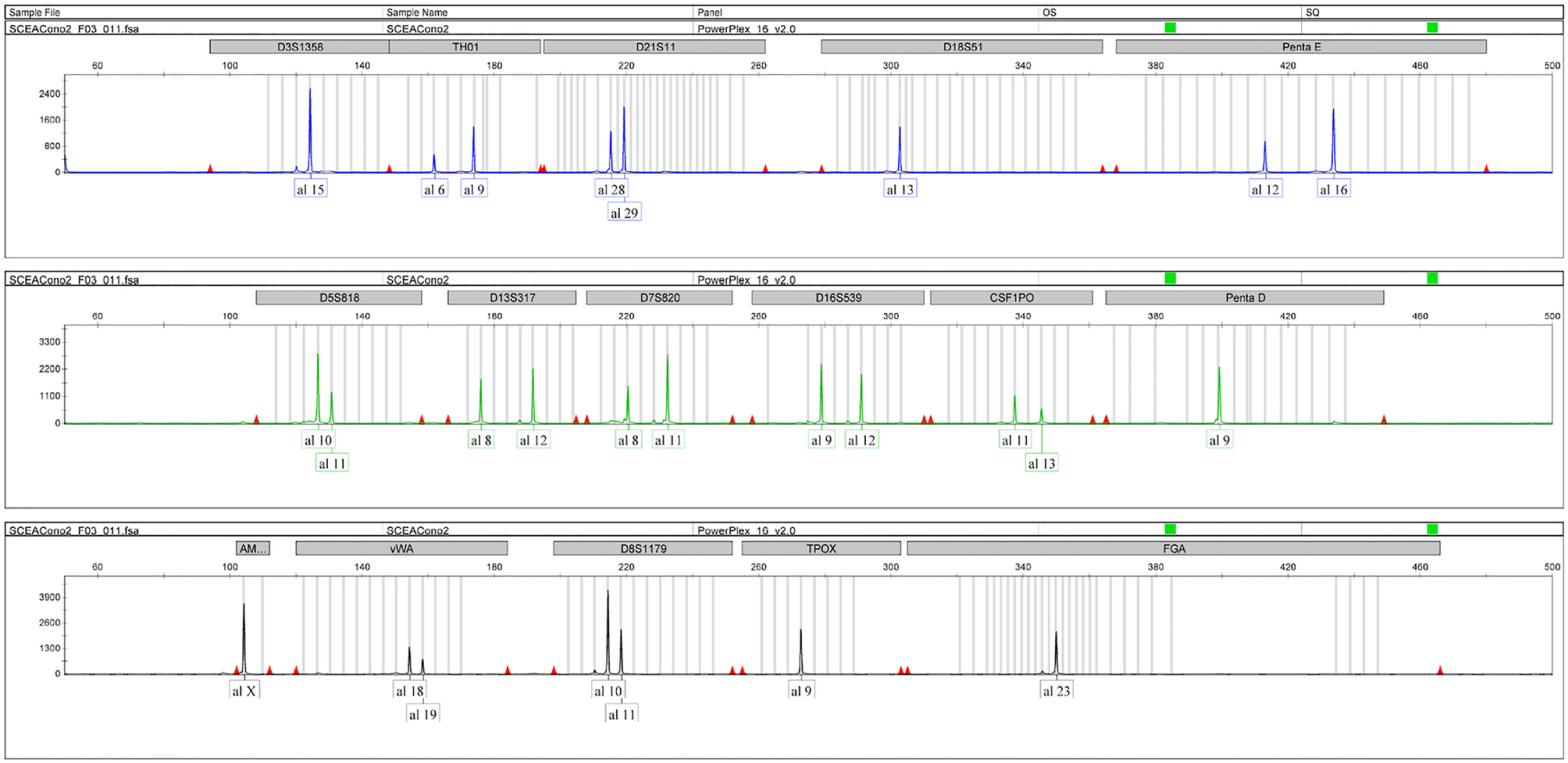
Short tandem repeat (STR) profiles. Short tandem repeat values of cell line and tumor tissue for fifteen gene loci and amelogenin (X and Y chromosomes). The evaluation value between the cell culture and host tumor tissue was 0.96, which was high enough that the STR profile of the cell line was the same as those of the host tumor sample.

To rule out the possibility of cross-contaminations with available cell lines, we performed STR analysis of SCEACono2. We have extracted the genomic DNA from the SCEACono2 cells after 100 times passaging. Although the STR profile of the cultured SCEACono2 cells did not exactly match that of the original patient tumor sample, their STR similarity was 0.96, indicating that the cultured SCEACono2 highly resembles the original tumor cells. (Fig. 5 and Table 1) We, therefore, considered these cells to be identical cell strains. In addition, we confirmed that there were no other identical cell lines in the Japanese Collection of Research Bioresources (JCRB).

**Table 1:** STR profile.

Locus	SCEACono2		Original tumor tissues	
D3S1358	15		15	
TH01	6	9	6	9
D21S11	28	29	28	29
D18S51	13		13	14
Penta_E	12	16	12	16
D5S818	10	11	10	11
D13S317	8	12	8	12
D7S820	8	11	8	11
D16S539	9	12	9	12
CSF1PO	11	13	11	13
Penta_D	9		9	
AMEL	X		X	
vWA	18	19	18	19
D8S1179	10	11	10	11
TPOX	9		9	11
FGA	23		23	

### Tumorigenicity in Nude mice

**Figure 6:**
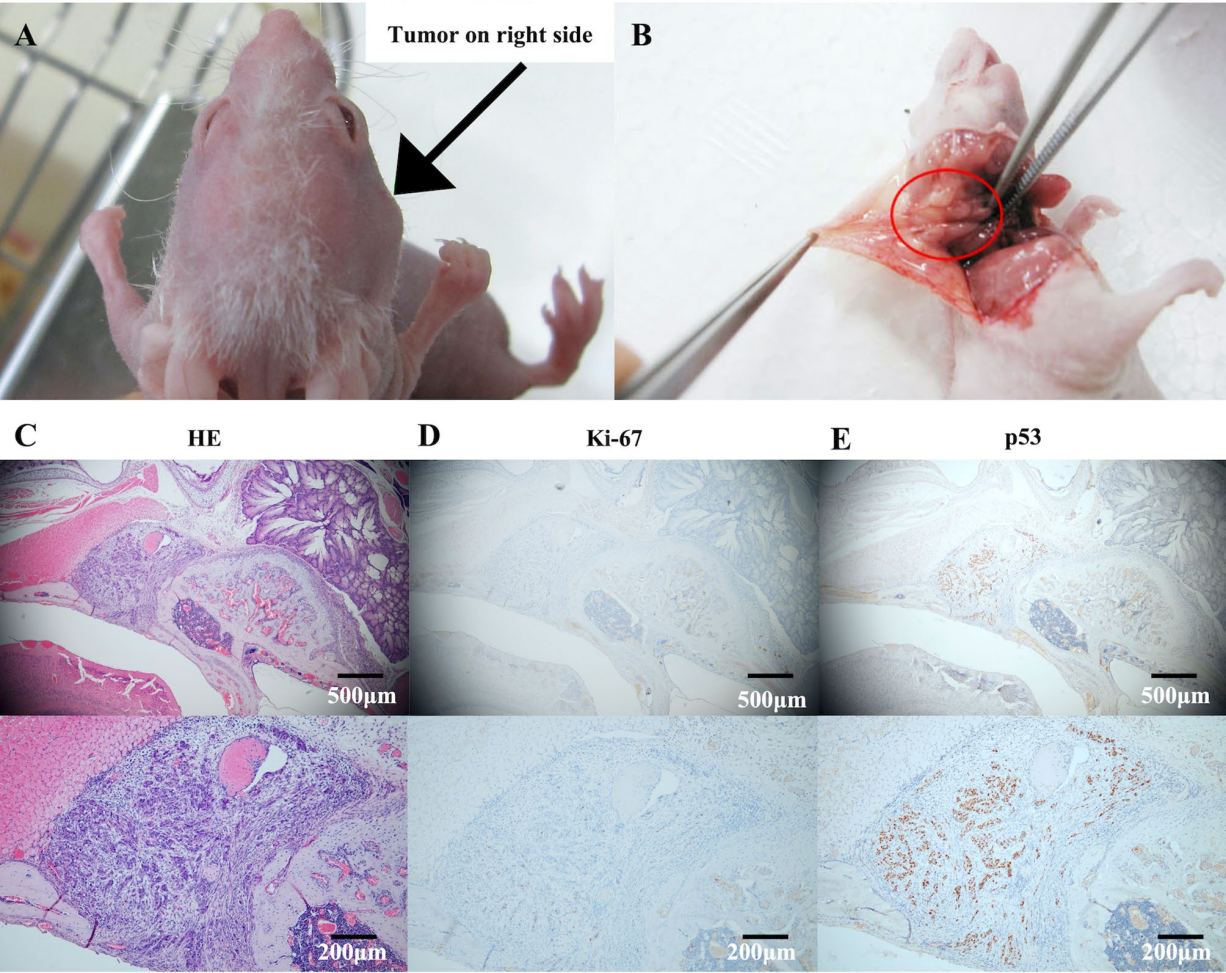
Tumorigenicity in BALB/c mice and histological analysis of the Cell line-derived Xenograft tumors in Nude mice. (**A**, **B**) Subcutaneous tumor mass (black arrow in **A** and red circle in **B**) in BALB/c mice which inoculated with 5 × 10^6^ cells at 14 days post-injection. (**C**) Hematoxylin and Eosin (H&E) stain of the paraffin-embedded tumor sample confirmed the tumor formation of the differentiated squamous cell carcinoma. (**D**, **E**) Immunohistochemistry (IHC) analysis of SCEACono2 xenografts. Human Ki 67 and p53 were detected by IHC. The slide IHC image under low magnification and representative IHC image under high magnification of p53 and Ki 67 stain were shown on the left and right, respectively.

We next examined the tumorigenic capacity of SCEACono2 in vivo. One week after the injection of SCEACono2 cells at the right temporal region in nude mice, subcutaneous tumor size was macroscopically increased, and we resected the tumors 16 days after inoculation. (Fig. 6A, B) We examined various injection sites to validate tumorigenicity of SCEACono2, including subcutaneous groin, knee, flank, and temporal sites. Importantly, the tumor formation rate of SCEACono2 exhibited 100% (n = 5), indicating that this cell line is suitable for cell line-derived xenograft models. Immunohistochemical staining showed that the xenografted tumor tissues were positive for the proliferation marker Ki 67 and p53 (Fig. 6C–E).

### Whole exome sequencing (WES)

To clarify the genetic alterations of SCEACono2, we performed WES on genomic DNA derived from the cultured SCEACono2 cells, the original tumor tissues and the patient-matched PBMC as the non-cancerous control. The sequencing data had an average sequencing depth of 126.16 (range; 124.39–127.89), suggesting sufficient quality for mutation calling. Cultured SCEACono2 cells harbor several non-synonymous somatic mutations such as *FREM3*(p.S61R), *GLIS3*(p.S24R), *COL15A1*(p.V1265E), *NOTCH1*(p.A465T), *FSCB*(p.A310V), *INF2*(p.R87H) and *TP53*(p.G245S) (Table 2). Among these mutations, *TP53* was also found as a significant mutation in the primary tumor. Although other mutations were excluded upon filtering, we confirmed that all of them were included in the primary tumor sample with low variant read frequencies (data not shown), suggesting that the cells harboring these mutations were selected among the heterogeneous cancer cell population. Taken together, SCEACono2 resembles genetic characteristics of the primary tumor.

**Table 2:** Newly established cell line harbor several somatic mutations, missense mutations.

Gene	Position	Chromosome	Start	End	CDS mutation	Amino acid change	Variant type
*FREM3*	4q31.21	4	144,621,646	144,621,646	c.C183A	p.S61R	Missense
*GLIS3*	9p24.2	9	4,286,354	4,286,354	c.T72G	p.S24R	Missense
*COL15A1*	9q22.33	9	101,829,306	101,829,306	c.T3794A	p.V1265E	Missense
*NOTCH1*	9q34.3	9	139,412,252	139,412,252	c.G1393A	p.A465T	Missense
*FSCB*	14q21.2	14	44,975,262	44,975,262	c.C929T	p.A310V	Missense
*INF2*	14q32.33	14	105,167,962	105,167,962	c.G260A	p.R87H	Missense
*TP53*	17p13.1	17	7,577,548	7,577,548	c.G337A	p.G245S	Missense

### Transcriptomic characteristics of SCEACono2

**Figure 7:**
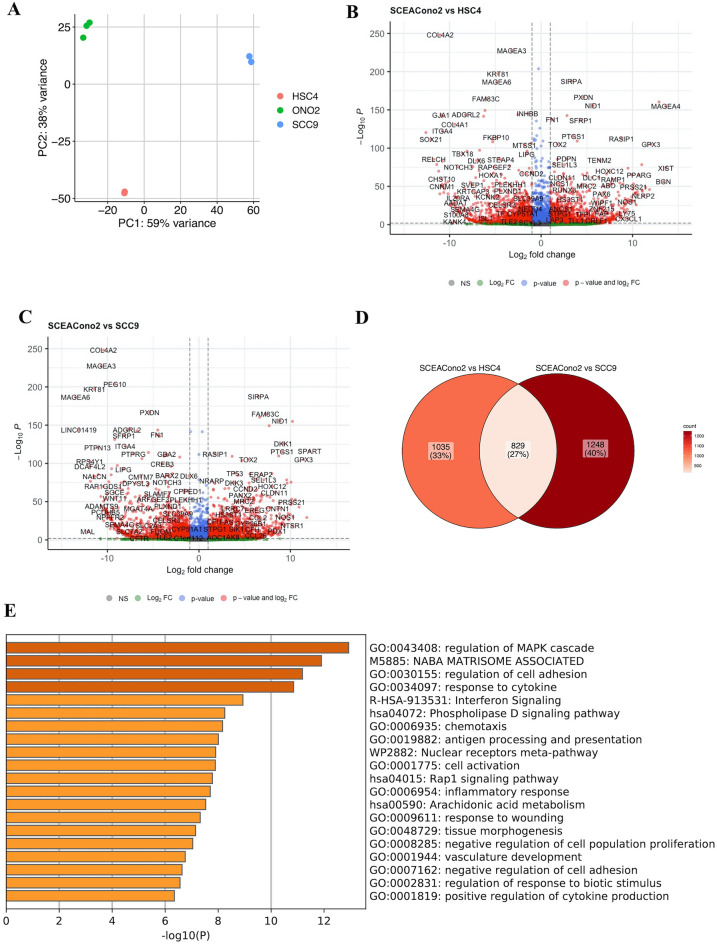
Comparison of gene expression among the newly established cell line, HSC-4, and SCC-9. (**A**) Principal component analysis showed the clear separation of these three cell lines in transcriptomic levels. (**B**) Volcano plot showing gene expression (FDR < 0.05; Log2FC > 1) altered (highlighted in red color) on the comparison between the SCEACono2 cell and HSC-4 cell. (**C**) Volcano plot showing gene expression (FDR < 0.05; Log2FC > 1) altered (highlighted in red color) on the comparison between the SCEACono2 cell and SCC-9 cell. (**D**) Venn diagram showing commonly upregulated genes in both comparisons of gene expression between the SCEACono2 cell line and HSC-4 and between the SCEACono2 cell line and SCC-9. Genes highlighted in pink are observed to be commonly upregulated in both comparisons. (**E**) Gene ontology (GO) enrichment analysis of upregulated genes. [− log10(*p* value)] of the corresponding biological process is shown. A dark orange bar indicates a small *p* value (*p* value < 0.1).

Finally, we performed RNA-seq and downstream bioinformatics analysis to explore the gene expression signature of SCEACono2 cells. Publicly available HNSCC cell lines HSC-4 and SCC-9 were subjected to this analysis simultaneously in order to compare their transcriptomic characteristics. Principal component analysis showed the clear separation of these three cell lines in transcriptomic levels, suggesting the unique transcriptional programs activated in SCEACono2 compared to HNSCC cell lines (Fig. 7A). We identified 1864 and 2077 upregulated genes in SCEACono2 compared to HSC-4 and SCC-9, respectively (Fig. 7B, C). In order to identify a unique transcriptional signature characterizing SCEACono2, we have extracted overlapped genes among these upregulated genes. 829 genes (27%) were found to be overlapped, whereas 1035 (33%) and 1248 (40%) genes were upregulated only in the comparison to HSC-4 and SCC-9 cell, respectively (Fig. 7D). Gene ontology analysis showed significant enrichment of genes involved in the regulation of MAPK cascade (GO: 0043408), regulation of cell adhesion (GO: 0030155), response to cytokine (GO: 0034097) and inflammatory response (GO: 0006954) in these overlapping upregulated genes. (Fig. 7E).

## Discussion

Squamous cell carcinomas of the head and neck include the tongue, pharyngeal, laryngeal, and sinonasal carcinomas. The development of treatment strategies for head and neck squamous cell carcinoma has been supported by building evidence from clinical and basic research. Temporal bone squamous cell carcinoma is included as a type of temporal bone squamous cell carcinoma. However, temporal bone squamous cell carcinoma of the external auditory canal and middle ear is extremely rare compared to other head and neck SCC, and the frequency of this pathogen has been reported to account for < 0.2% of head and neck squamous cell carcinoma^1,4,18^. Due to its rarity, the accumulation of molecular biological evidence and establishing of treatment guidelines have been delayed. The immortalized cell lines for each pathogen have played a major role in establishing molecular biological evidence, elucidating molecular biological mechanisms of carcinogenesis and metastasis, or verifying therapeutic resistance and radiosensitivity. The establishment of cell lines for squamous cell carcinoma of the external auditory canal has lagged far behind that of other head and neck SCC. We have previously reported a method for creating a primary culture of cancer cells isolated from patient cancer tissue. Using this method, we have established a new cell line of squamous cell carcinoma of the external auditory canal. Therefore, in this report, we verified and reported the molecular and biological characteristics of the new cell line.

According to the Cellosaurus (https://web.expasy.org/cellosaurus/), excluding derivative cell lines, only one cell line, CAE606, is available as a cell line derived from the external auditory canal squamous cell carcinoma so far^12^. As we have previously reported, maintaining cancer cells isolated from squamous cell carcinoma of the external auditory canal is still challenging; the rarity of the disease and the difficulties in maintenance of the primary cells may explain why the establishment of immortalized cells from this disease has rarely been reported. Furthermore, the process of cell line establishment from fresh tumor tissues is energy- and time-consuming, and difficult due to contamination, out-growth of cancer-associated fibroblasts and cellular senescence, which causes the low success rate of establishment^19^. In this study, we tried to establish the new cell line from external auditory carcinoma based on the method that our group had reported previously^13^. We did not use either SV40 T-antigen, HPV E6/E7 or telomerase reverse transcriptase to immortalize cells^20–25^. During the primary cell culture, we could obtain a colony with enough mutations of the intrinsic gene to result in long-term survival, and our newly established cell line without the transfer of exogenous genes will be more suitable for studying EAC-SCC^26^.

The shape of SCEACono2 cells was quite similar to the cobblestone-like epithelioid morphology. The population doubling time was about 24 h, and this proliferative capacity can be comparable to other cells, including the Hela and SCC-9, commonly used in scientific research. Our newly established cell line harbors the typical markers of squamous cell carcinoma, including the SCCA1/2 and p53. Furthermore, this cell line expresses both vimentin and cytokeratin. This is implicated in the epithelial mesenchymal transition, associated with invasive behavior, poor differentiation, and poor prognosis. Several cancer stem cell markers, CD44, CD133, and Oct3/4, were positive, and ALP was partially positive^14–17,27–29^. These findings mean that this cell line has the characteristic of the stem cell partially. The SCEACono2 cells also displayed chromosomal instability, including the changes in chromosome number and the generation of hybrid DNA, which is related to the promotion of tumorigenesis and development^30–33^.

We confirmed that this new cell line could be a tool for scientific research in vivo. Injection of the newly derived cells under the temporal skin of a nude mouse resulted in tumor formation. The immunohistochemistry staining of xenograft shows the positive of Ki 67 and p53. This cell-line-derived xenograft could be used to elucidate the tumor's anatomical metastatic mechanism and select therapeutic agents for EACSCC.

In this study, we analyzed the transcriptome of the newly established cell line with RNA sequencing compared to two cell lines of tongue squamous cell carcinoma: HSC-4 and SCC-9. We found that the genes related to inflammation and cell adhesion are more expressed in the newly established cell line than the other two. Furthermore, newly established cell line harbor several somatic mutations: *FREM*(p.S62R)*, GLIS3*(p.S24R)*, COLI5A1*(p.V1265E)*, NOTCH1*(p.A465T)*, FSCB*(p.A310V)*, INF2(*p.R87H)*, and TP53*(p.G245S). The somatic mutation of TP53 is the most frequently mutated tumor suppressor gene in human squamous cell carcinoma^34^. Furthermore, The somatic mutation of *NOTCH1* is also commonly found in human squamous cell carcinoma^35^. These results suggest that the major drive mutations of EACSCC are similar to those of other SCC. In addition to these somatic mutations, missense mutations in *FREM3, GLIS3, COLI5A1, FSCB, and INF2* are identified. However, the molecular significance of these missense mutations in EACSCC remains unclear. The previously established cell line, CAE606, harbors inactivating mutation in *CDKN2A* and *TP53* and high-level amplification of *CCND1*, affecting the cell cycle pathway. Our cell line also harbors the major drive mutations of EACSCC and has the transcriptome profiling related to inflammation and cell adhesion. These analyses with next-generation analysis suggested that this newly established cell line with these genetic backgrounds is an appropriate cell line for EACSCC research.

In conclusion, we established new cell lines derived from human squamous cell carcinoma of the external auditory meatus and named SCEACono2. This newly established cell line could be a useful tool for the pathological analysis of EACSCC to provide further insight into the mechanism of tumorigenesis, metastasis, and selection of the antitumor-drug.

## Methods

### Ethic statement

The Clinical Research Ethics Review Committee of Kyushu University Hospital approved the study (permit no. 29-43, 30-268, and 700-00). Written informed consent for the current research project was obtained before the tumor tissue, and a blood sample were harvested. This study was also conducted according to the principles of the Declaration of Helsinki. The animal experiment was also reviewed and approved by the Committee of. Ethics of Animal Experiments, Kyushu University Graduate School of Medical Sciences. The animals were treated according to the “Guidelines for Proper Conduct of Animal Experiments” (Science Council of Japan). The study was carried out in compliance with the ARRIVE guidelines.

### Growth medium

The growth medium consisted of Dulbecco’s Modified Eagle Medium (D-MEM)/Ham’s F12 (Sigma-Aldrich, St. Louis, MO), supplemented with 10% fetal bovine serum (FBS: Sigma-Aldrich), 0.1 mM MEM Non-Essential Amino Acids Solution (Thermo Fisher Scientific, Waltham, MA), 1 mM sodium pyruvate (Thermo Fisher Scientific), 2 mM L-glutamine (Thermo Fisher Scientific) and 1% Antibiotic–Antimycotic Mixed Stock Solution (Nacalai Tesque, Inc., Kyoto, Japan).

### Immunofluorescence and alkaline phosphatase staining

SCEACono2 cell for immunostaining was prepared as follows. SCEACono2 suspension for staining the Vimentin, Cytokeratin, p53, SCCA1/2, CD133, Oct3/4, and CD44 were seeded on eight-chamber slides and allowed to attach overnight. The cultures were fixed with methanol: acetone 1:1 (v/v) for 20 min at − 20 °C. These preparations for cytoplasmic antigen staining were additionally permeabilized with PBS containing 0.5% Triton X-100 and 0.05% NaN3 for 10 min at r.t. Following fixation, for cell surface marker staining, preparations were incubated with anti-CD44-PE or anti-CD133-APC for 1 h at r.t. For cytoplasmic marker staining, samples were incubated with anti-pan-Cytokeratin-Alexa Fluor® 488 and Vimentin-PE at r.t. for 1 h and with anti-p53-Alexa Fluor® 488 and anti-SCCA1/2 (mouse IgG) at 4 °C overnight. For SCCA1/2 and Oct3/4 antigens, the samples were stained with goat anti-mouse IgG (H + L) highly cross-adsorbed secondary antibody conjugated Alexa Fluor® 555 after the primary antibody reaction. Slides were then mounted with DAPI Fluoromount-G® (SouthernBiotech, Birmingham, AL) for nuclear staining. Unfixed cytospin preparations were tested with the Vector® Blue Alkaline Phosphatase Substrate kit (Vector Labs, Burlingame, CA) to check for alkaline phosphatase (ALP) activity.

### Chromosome analysis: chromosomes of SCEACono2 cell

After 48 h of incubation in a culture medium, Semiconfluent and actively dividing cells were incubated in 0.5 μg/ml Colcemid for 20 min, and cells were harvested. Harvested cells were pelleted out and treated with hypotonic solution (0.075 M KCL) for 10 min before fixation in methanol-acetic acid (3:1). The suspension of fixed cells was dropped onto microscope slides. Chromosomes of SCEACono2 cells were prepared using a standard air-drying method, metaphase spreads were counted to determine the modal number, and the G-banding technique analyzed karyotypes. The metaphase chromosome spreads slide was evaluated using Ikaros software (MetaSystems, Altussheim, Germany).

### Cell-line-derived xenograft

In vivo, the tumorigenicity of the SCEACono2 cells was assessed by the ability to form tumors subcutaneously in Nude mice. SCEACono2 cells were harvested and re-suspended to prepare a cell suspension of 5 × 10^7^ cells/ml. BALB/c nude mice (5 weeks, body weight 16–18 g) were subcutaneously injected in a 0.1 ml suspension in each region. When the apparent tumor formation was macroscopically confirmed, the tumor was separated from the mice and fixed in 4% formaldehyde for pathological examination. The tumorigenesis experiment was repeated using 3 Nude mice. BALB/c nude mice were purchased from The Jackson Laboratory Japan, Inc. The xenograft tumors were examined with immunohistochemistry measure. Following deparaffinization, hydration, and blockage of endogenous peroxidase activity by 3% hydrogen peroxide, the specimen sections underwent antigen retrieval process in citrate buffer (pH6.0) and were incubated overnight at 4 °C with antibodies against p53(1:800, NCL-L-p53-DO7, Leica Camera AG, Wetzlar Germany) and Ki 67(1:100 M7240, Dako, Carpinteria, CA, USA). Then the sections were incubated with poly-peroxidase-anti-mouse/rabbit IgG and stained with diaminobenzidine (Fujifilm Wako Pure Chemical, Osaka, Japan), followed by counterstaining with hematoxylin and dehydration.

### Short tandem repeat analysis

Short tandem repeat analysis Genomic DNA was extracted from the cell line and the tumor samples from the patients using a DNeasy Blood & Tissue Kit (Qiagen, Valencia, CA, USA). The Gene Print PowerPlex 16HS System (Promega, Madison, WI, USA) was used to amplify the extracted DNA. The amplified fragments were detected using an Applied Biosystems 3500xL Genetic Analyzer (Thermo Fisher Scientific).

### DNA/RNA extraction, library preparation

The extraction of genomic DNA from the SCEACono2 and the patient-matched PBMC was conducted using DNeasy Blood and Tissue Kits (Qiagen, Chatsworth, CA, USA). The extraction of RNA from SCEACono2, HSC-4, and SCC-9 cell lines were performed using a NucleoSpin™ RNA plus kit (MachereyHagel, Düren, Germany).

### Whole exome sequencing (WES)

Library preparation from DNA extracted from cultured SCEACono2 cells, the parent EACSCC tissue as well as patient-matched PBMC was conducted using an Agilent SureSelect All Exon V6 exome capture kit (Agilent Technologies, Santa Clara, CA, USA). Library preparation and WES were performed at the Beijing Genomics Institute (Shenzhen, China). The captured libraries were sequenced using DNBSEQ-G400 sequencer with the paired-end 100-bp sequence read option according to the manufacturer’s protocols.

### Alignment and detection of somatic variants of SCEACono2 cells

The WES data was processed in the Genomon 2.6.2 pipeline (http://genomon.hgc.jp). Briefly, the sequencing reads were aligned to the NCBI Human Reference Genome Build 37 (hg19) with BWA version 0.7.8 using default parameters (http://bio-bwa.sourceforge.net/). PCR duplicate reads were removed using Picard (http://www.picard.sourceforge.net). Detection of somatic mutations in SCEACono2 cells and the parent EACSCC tissue was performed using the EBCall algorithm (Shiraishi Y, Sato Y, Chiba K, et al. An empirical Bayesian framework for somatic mutation detection from cancer genome sequencing data. Nucleic Acids Res. 2013;41:e89.) with the following parameters: (a) mapping quality score ≥ 30; (b) base quality score ≥ 15; (c) sequencing depths ≥ 8; (d) variant reads in SCEACono2 and EACSCC ≥ 4; (e) variant allele frequency (VAF) in SCEACono2 and EACSCC ≥ 0.05; (f) VAF in PBMC < 0.1; (g) minus logarithm of P-value of Fisher’s exact test ≥ 1.3; and (h) minus logarithm of the *P* value of EBCall ≥ 5.

### RNA sequencing and downstream analysis

Library preparation from RNA extracted from cultured cells and RNA sequencing were performed using a DNBSEQ-G400 sequencer at the Beijing Genomics Institute (Shenzhen, China). The sequenced reads were aligned to the human reference genome GRCh38 using STAR v2.7.9a with Gencode v38 annotations. Gene count tables were generated with RSEM v1.3.1. Differentially expressed genes (DEGs) between SCEACono2, HSC-4 and SCC-9 was detected using R package DEseq2 v1.10.1. Cut-off values of DEGs were determined as absolute log2 fold change > 2 and FDR-adjusted *p* value < 0.01. Gene ontology analysis was performed using Metascape.

### Informed consent

Informed consent was obtained from all subjects involved in the study.

### Supplementary Information

Supplementary Information 1.

Supplementary Legends.

## Data Availability

The RNA-Seq datasets (accession no. GSE240311) and the whole exome sequencing data (accession no. JGAS000645) have been deposited to the NCBI Gene Expression Omnibus (https://www.ncbi.nlm.nih.gov/geo/) and the Japanese Genotypephenotype Archive (https://www.ddbj.nig.ac.jp/jga/index.html), respectively.
