# Supplementary figures and images for: Biological and genetic characterization of a newly established human external auditory canal carcinoma cell line, SCEACono2
Source: Sci Rep. 2023 Nov 10;13:19636. doi: 10.1038/s41598-023-46926-y (PMC10638439; doi:10.1038/s41598-023-46926-y)

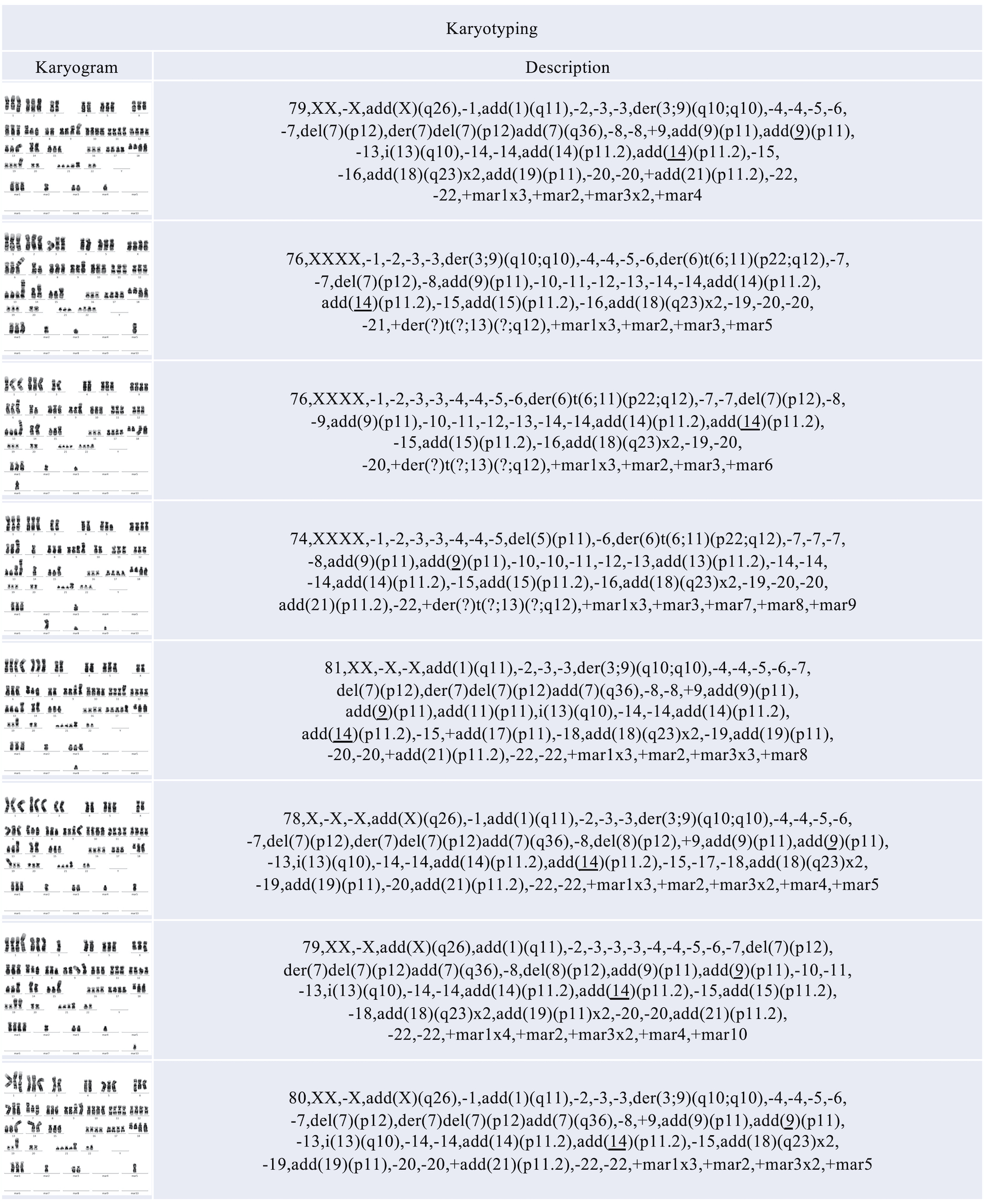

Supplement: Supplementary file 1 — Supplementary Information 1. [file 41598_2023_46926_MOESM1_ESM.jpg]
