# Supplementary material for: Biological and genetic characterization of a newly established human external auditory canal carcinoma cell line, SCEACono2
Source: Sci Rep. 2023 Nov 10;13:19636. doi: 10.1038/s41598-023-46926-y (PMC10638439; doi:10.1038/s41598-023-46926-y)
Supplement: Supplementary file 2 — Supplementary Legends. [file 41598_2023_46926_MOESM2_ESM.docx]

Supplementally figure 1: Chromosome analysis of SCEACono2 cells. karyotyping analysis was also performed on 8 cells that could be analyzed. Eight cells showed abnormal karyotypes, indicating chromosomal instability.
